# Examining the Effectiveness of Gottman Couple Therapy on Improving Marital Adjustment and Couples' Intimacy 

**Published:** 2018-04

**Authors:** Maryam Davoodvandi, Shokouh Navabi Nejad, Valiollah Farzad

**Affiliations:** 1 Department of Counseling, Science and Research Branch, Islamic Azad university, Tehran, Iran.; 2 Department of Counseling, Kharazmi University, Tehran, Iran.; 3 Department of Psychology, Islamic Azad University of Central Tehran Branch, Tehran, Iran.

**Keywords:** *Couples' Intimacy*, *Gottman’s Couple Therapy*, *Marital Adjustment*

## Abstract

**Objective:** The present study aimed at examining the effectiveness of Gottman couple therapy on improving marital adjustment and couples' intimacy.

**Method** **:** This was a semi- experimental study with pretest, post-test, and follow-up assessments. A total of 16 couples (32 individuals) were selected using convenience sampling method considering inclusion- exclusion criteria; they were then randomly assigned into experimental (N = 16) and control (N = 16) groups. Participants of the experimental group received ten 45-minute sessions of Gottman’s couple therapy. The research tools were Spanier Questionnaire and Walker and Thompson’s Questionnaire. Data were analyzed using Mixed design MANOVA.

**Results:** Findings revealed that Gottman’s couple therapy approach had positive effects on improving marital adjustment (P = 0/001) and couples' intimacy (P = 0/001). Furthermore, the results of assessments in the follow-up period indicated that Gottman’s couple therapy had enduring effects on marital adjustment and couples' intimacy.

**Conclusion: **According to the results of the present study, Gottman method can be used as an effective treatment to improve marital relationships, adjustment, and intimacy. Therefore, researchers, therapists, and other authorities should pay particular attention to this method.

In the last 50 years, cultural changes have had a significant impact on couples' relationships and the structure of families (1). These structural changes, which are the results of cultural transformation, can be one of the effective factors in the growing trend of divorce (2). Statistics released by various communities indicate an increase in the separation of couples; for example, according to the American Psychological Association (2017), 40% to 50% of American couples divorce annually (3). The rate of divorce has also been growing in Iran in recent years. According to statistics provided by the Organization for Civil Registration, 25% of marriages that took place from March to December 2015 have led to divorce, and the rate of divorce had an ascending trend, almost 3% annually (4). 

Therefore, the preservation and promotion of family relationships requires evaluation of the stability and quality of marital relationships through evaluating such concepts as consistency, intimacy, and success (5). In this regard, couple’s adjustment is one of the variables that can affect the quality of marital conflict. Accordingly, Lata Rao (2017) believes that marital adjustment is an adaptive behavior through which couples meet the needs of each other. During this 

process, spouses learn how to cooperate with each other in different fields over time and adjust themselves with the circumstances (6). 

In addition, Byrne, Carr, and Clark (2004) concluded that marital adjustment is a process with 4 aspects of marital functioning including marital satisfaction, solidarity, consensus and cohesion, and expression of emotions and feelings of couples in the family environment (7). Therefore, marital adjustment is a psychological situation that does not arise by itself, rather, its acquisition requires the efforts of couples and learning some skills. 

Among the most important of them is the speaker-listener technique of the Gottman approach that was applied in a study by Coie et al. (1993) and caused significant improvement in positive emotions, better relationship, and problem-solving behaviors (8). 

Usually cited in the literature, couple’s intimacy is another important component necessary for improving marital relationship. As the means of exchange and mutual satisfaction of emotional and psychological needs in an acceptable and anticipatory way, intimate relationships between couples can also strengthen the affective relationship and marital satisfaction (9, 10). According to Yoo, Bartle, Day and Gangamma (2014), modification of couple’s communications through controlling negative emotions and reducing marital conflicts results in improved emotional intimacy of couples (11). Moreover, Khojasteh Mehr, Soodani, Ahmadi Ghozlojeh, and Shiralinia (2015) showed that couples therapy can increase couple’s emotional intimacy (12).

Research evidence suggests that increased adjustment and intimacy among couples can play an important role in marital satisfaction and common life stability (13). Hence, identifying therapeutic strategies affecting these dimensions is always of concern to family therapists. In this regard, Gottman is currently a pioneer researcher in the field of couple therapy (14). Gottman's approach (1977) is an integrated approach that has been used as a fundamental principle of various therapeutic theories, such as systems theory, existential point of view, and narrative therapy (15). Gottman's method also follows psychoanalytic point of view and supports behavioral approaches because it seeks behavior change (16). Gottman considers effective relationship as the most important skill for couples and believes that those with effective relationship have the ability to reach mutual approval and listen to their spouse's needs and respond to them non-defensively; and when there is a misunderstanding, they focus on the problem and establish a peaceful relationship (17). Several studies have been conducted regarding the Gottman approach. Gottman et al. (2013) evaluated the effect of short-term psychological training on couples and showed its positive impact on 3 variables of satisfaction with relationship, quality of friendship, and destructive conflict in a one-year follow-up (18). Moreover, in other studies based on the Gottman theory, the results indicated that Gottman couple therapy was effective in reducing emotional divorce (19), reducing stress and increasing marital happiness (20). 

Given the novelty of the Gottman approach and the limited research done in this context, especially on the variables of marital adjustment and intimacy, the present study aimed at determining the effectiveness of Gottman approach and the stability of its effect on marital adjustment and couple’s intimacy, using experimental Gottman couple therapy group and a control group.

## Materials and Methods

This was a quasi-experimental study with pretest, posttest, one-month follow-up, and control group. The statistical population included all couples referring to the counseling center of Department of Education, District 9 of Tehran. A total of 16 couples were selected using convenience sampling method according to inclusion and exclusion criteria and were randomly assigned into experimental and control groups. Inclusion criteria were as follow: (1) a minimum of high school diploma, (2) not on the verge of divorce, (3) commitment to participate in the therapy sessions, (4) having children of school age, and (5) a low score in the Spanier Dyadic Adjustment Scale and in the Walker and Thompson Intimacy Scale. Exclusion criteria were the use of other psychological treatments and addiction of any of the spouses.

**Spanier Measure Scale (DAS):** This scale, which was first developed by Graham Spanier in 1976, includes 32 items and is used to assess the quality of marital relationship (21). The total adjustment in a sincere relationship can be evaluated by calculating the total score. The total score ranges from 0 to 150, with the higher scores indicating better adjustment (22). The scale Cronbach’s alpha is 0.96, and its retest reliability is reported to be between 0.70 and 0.95 (21). This scale was revised by Busby et al. (1995) and the internal consistency of the satisfaction, cohesion, consensus, and affectional expression dimensions was reported to be 0.94, 0.81, 0.90, and 0.73, respectively (23). In Iran, Shahi (2000) determined the validity of this scale for the first time based on the correlation of this questionnaire with the Locke & Wallace adjustment questionnaire (0.85) (24, 25). The reliability of the scale was reported by Hosseini (2011) as 0.91 (26). In the present study, the reliability of the scale was obtained to be 0.92 through Cronbach’s alpha.

**Intimacy Questionnaire by Walker & Thompson:** This scale was developed by Walker and Thomson in 1983 and consists of 17 items. The subject’s score is obtained by adding the scores of items and dividing them by 17. Scores range from 1 to 7, and the higher score reflects higher intimacy. Walker and Thompson (1983) reported the reliability of this test as 0.91-0.97 using Cronbach’s alpha (27). Scale validity was also determined through content and face validity (22). This scale was first translated and validated in Iran by Sanaei (2000). The reliability of the intimacy scale was found by Hosseini (2011) as 0.86 using Cronbach’s Alpha (26). In the present study, the reliability of this scale was found to be 0.88% through Cronbach’s alpha.

*Procedures*

In the first stage, 16 couples (32 individuals) were selected from the clients introduced by the counseling center after the interview and according to inclusion and exclusion criteria. Informed consent was obtained from all participants. Finally, all participants were randomly assigned into 2 groups (experimental Gottman’s couple therapy and control), each with 8 couples (16 individuals). The participants were assessed using the Dyadic Adjustment and Intimacy Scales in 3 stages of pretest, posttest, and 2-month follow-up. The experimental group received 10 sessions of Gottman’s couple therapy (pairwise, not in group), while the control group was placed in the waiting list. Treatment sessions were performed once a week for 90 minutes. As observed int table 1, the framework of treatment sessions based on Gottman's method is presented, that was taken from the model of House Sound Relationship (28).

## Results

Data were analyzed with mixed analysis of variance according to the 3 measurement times. To this end, the M Box test was used to ensure observation of this test assumption (i.e., the equality of variance-covariance matrices across the cells was formed by the between- subject's effects), which revealed that the null hypothesis of this assumption was rejected (Box M = 54.17, p<0.05). However, given the equal size of sample groups, mixed design analysis of variance is robust to violation to this assumption. According to the Wilks’ Lambda (0.97; p<0.05, F (4, 27) = 130.433, Eta square = 0.95), it was found that the measurement time interacting with the 2 experimental groups had a significant impact on the linear combination of dependent variables. Then, the assumption of sphericity was implemented for all variables using Muchley test, and the results showed that this assumption was not observed (p<0.05, W Mauchly’s compatibility = 0.750). Therefore, the Greenhouse-Geisser test was used to modify the degrees of freedom in analysis of variance. The results indicated the significance of the measurement time effect (pretest, posttest, follow-up) on the adjustment variable according to the group membership type of the couples (F _(1.6, 48.010)_ Greenhouse-Geisser=334/14, p<0.05). In addition, regarding the intimacy component, the results of Mauchly test showed compliance with this assumption (W Mauchly’s intimacy = 0.928, p<0.05), indicating no need to modify the degrees of freedom in the analysis of variance. The results of analysis of variance with the sphericity assumption showed a significant difference between marital intimacy of the experimental and control groups in the 3 measurement times (pretest, posttest, follow-up) (p<0.05, F (2, 60) sphericity assumption = 77/75).

**Figure 1:**
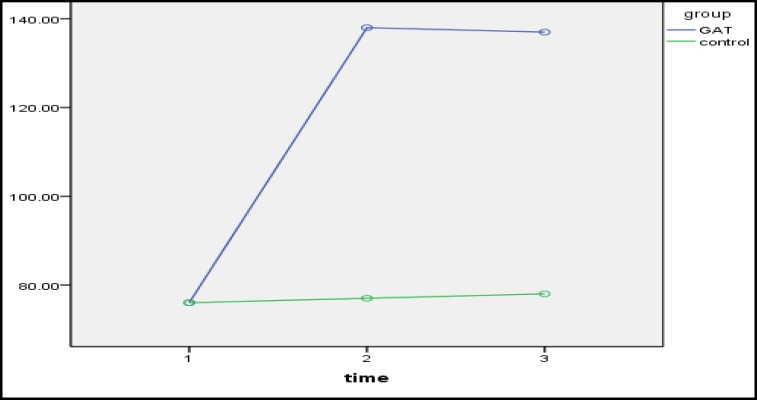
The Interactive Effect of Time-Group to Evaluate the Effectiveness of Intervention in Adjustment

**Figure 2:**
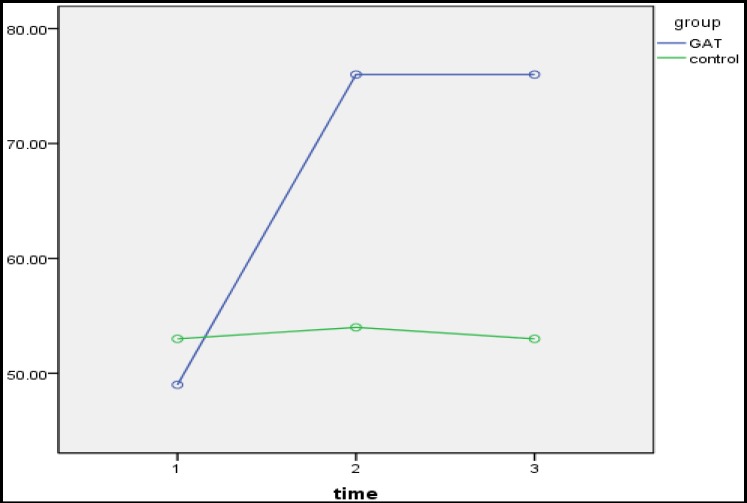
The Interactive Effect of Time-Group to Evaluate the Effectiveness of Intervention in Intimacy

As observed in Table 2, a significant difference existed between adjustment and intimacy of couples in the treatment and control groups in the pretest-posttest (p<0.05). Moreover, the higher mean of the experimental group in the posttest represented the impact of intervention Gottman couple therapy on increasing marital adjustment (Figure 1) and intimacy (Figure 2). In addition, there was no significant difference in the posttest follow-up period (p>0.05), which represented the stability of the intervention effects.

**Table 1:** Intervention Modules and Component Summary Based on Gottman Method

**Session**	**Meetings Summary Framework**
First	Communicating with couples and evaluating
Second	Modifying map of love
Third	Strengthening the sense of attachment and praise
Fourth	Taking steps to each other instead of turning backs on each other
Fifth	Accept your partner’s influence
Sixth	Solving solvable problems
Seventh	Continuing to train the pattern for solving conflicts and remove obstacles and problems
Eighth	Goal of the sixth principle, overcoming the barriers of concept of impasse in marital relations, identifying the impasse causes
Ninth	The realization of common concept
Tenth	Final discussion regarding the meetings and posttest

**Table 2:** The Results of Within-Group Effect Test to Evaluate the Effectiveness of Intervention in Adjustment and Intimacy

**Variable**	**Type of ** **group**	**Pre-test**		**Post-test**		**Follow-up**		**Pretest- ** **post-test**		**Pre-test-** **follow-up**		**Post-test-** **follow-up**	
		**Mean**	**SD**	**Mean**	**SD**	**Mean**	**SD**	**p-** **value**	**Eta ** **Sq.**	**p-** **value**	**Eta ** **Sq.**	**p-** **value**	**Eta ** **Sq.**
Adjustment	Experimental	76.06	9.190	138.56	9.018	137.18	9.027	0.0001	0.94	0.0001	0.92	0.34	
	Control	76.87	7.482	77.68	10.460	78.18	9.361						
Intimacy	Experimental	49.81	9.020	76	6.683	76.43	9.639	0.0001	0.77	0.0001	0.77	0.37	
	Control	53.37	11.982	54.5	11.290	53.06	9.348						

## Discussion

This study aimed at investigating the effectiveness of Gottman couple therapy on improving marital adjustment and couple’s intimacy. Comparison of the results of the experimental and control groups indicated the effectiveness of Gottman couple therapy approach on improving marital adjustment and couple’s intimacy. The findings of this study are consistent with those of Shapiro, Gottman & Fink (2015) (29), Gottman, Coan, Carrere, Swanson (1998) (30), Babcock, Gottman, Ryan & Gottman (2013) (18), Gottman (1994) (31), Mahmoudi, et al. (2015) (32), and Moharrami (2013) (33).

In a study, using Gottman approach, Shapiro, Gottman, and Fink (2015) investigated the short-term variations of couple’s conflict in the period of transition to parental phase. Gottman approach interventions helped couples develop positive changes in their relationship 3 months after the end of treatment. During treatment, the offending behaviors of husbands decreased and positive feelings in women and men increased (29).

In another research, Gottman (1994) assessed more than 2000 couples in a longitudinal study and found that, on average, positive interactions and comments were 5 times more than negative interactions and comments in happy couples (31).

Therefore, in explaining the findings of this research, Gottman approach can be regarded as a method emphasizing the strengthening of the optimistic view to achieve greater compatibility. Increasing self-disclosure and the ability of mutual understanding to express interest and improve interactions were among the key principles in the implementation of treatment in this study. Finally, Gottman approach has a non-pathologic attitude that guides couples to find their capabilities to improve their relationship and solve problems, which will result in a favorable adjustment. 

In another study on conflicting couples, Gottman et al. (1998) found that positive affection was the best predictor of communication satisfaction and stability in newly married couples. Thus, Gottman approach increased couple’s intimacy and joy through helping them to engage in positive interactions and respect for each other’s ideas and to use proper methods of discussion (30).

The present study is also consistent with the research of Mahmoudi et al. (2015) and Moharrami (2013) who indicated that Gottman couple therapy improved satisfaction, adjustment, positive emotions, and marital intimacy, as well as all subscales of communication patterns and all subscales of marital conflict (32,33).

Therefore, Gottman’s Sound Relationship House model is a general plan to help deepen intimacy, manage conflict, and share what is meaningful for both couples. According to the theory of Gottman’s Sound Relationship House, this therapeutic approach, which influences the improvement of couple’s relationships, provides a constructive map for creating love and mutual understanding between couples. This approach is based on clinical practice performed on the stability of relationship and happiness of couples over more than 4 decades (14).

The first 3 levels of Sound Relationship House describe the system of friendship and intimacy. The “map of love” at the first level refers to the awareness and interest of the spouses to the inner world, thoughts, hopes, ideas, and feelings of each other. This level creates a sense of recognition. At the second level, paying attention to each other, as well as admiration and appreciation of the spouses create a sense of value and attention. In the third level, the spouses are helped to move towards each other instead of moving away from one another. This is the smallest unit of assessment of intimacy that is reflected in the effort of spouses to communicate with one another. As a result of this therapeutic process, an emotional bank account will be formed between couples. These efforts provide opportunities for improving communication. Therefore, improvement in intimacy in the present treatment can be attributed to the efforts made at the above 3 levels.

Small sample size and application of this therapeutic approach to those visited the counseling centers of the Department of Education in Tehran were among the limitations of this study. Moreover, lack of varied and available resources and research on Gottman method was another limitation of the present study. Hence, generalization and application of the results of this study should be done with caution.

## Conclusion

The results of the present study confirmed the effectiveness of Gottman's couple therapy on improving couples' intimacy and adjustment in studied statistical sample. In general, integrated treatment interventions seem to be appropriate for helping the couples with widespread, multidimensional and serious problems in their marital relationships. The Gottman Method for Healthy Relationships [as an integrated approach] helps couples to be able to manage marital relationships and develop problem-solving skills. These skills make couples more flexible in their relationships and help them achieve a high degree of emotional stability and a peaceful life. Therefore, the discussed changes will have a positive effect on marital relationships, compatibility, and intimacy among couples. According to the results of the present study, Gottman method can be used as an effective treatment in improving marital relationships, compatibility, and intimacy, which will result in increasing family strength. Therefore, researchers, therapists, and other authorities should attend to this theory. Moreover, to test the effectiveness of this therapeutic approach, it is recommended that this approach be tested in other statistical populations as well.
